# Perceived stress and physiological consistency during mental stress exercises and controlled breathing

**DOI:** 10.1192/j.eurpsy.2022.1895

**Published:** 2022-09-01

**Authors:** D. Vasquez, E. Mejia-Mejia, R. Torres, D. Restrepo

**Affiliations:** 1 Universidad de Antioquia, Grupo De Neurociencias De Antioquia, Medellin, Colombia; 2 University of London, University Of London, London, United Kingdom; 3 Escuela de Ingeniería de Antioquia, Ingeniería Biomédica, Medellin, Colombia; 4 Universidad CES, Facultad De Medicina, Medellin, Colombia

**Keywords:** Emotional stress, Physiological, Breathing exercises, Heart rate

## Abstract

**Introduction:**

The measurement of the physiological coherence, the order and the quality of the connection of complex systems such as the cardiac and the respiratory system, varies in situations of stress and relaxation.

**Objectives:**

We aim to assess changes in physiological coherence and perception of stress during mental stress and directed breathing exercises.

**Methods:**

Repeated-measures study in healthy adults without prior training in breathing techniques, aged between 18 and 65 years of both sexes who were evaluated in three situations: baseline, mental stress (Stroop test and successive subtractions), and directed breathing, during which were captured heart rate and respiratory signals to estimate physiological coherence and the participants rated the perceived stress at each moment.

**Results:**

34 participants were analyzed, 59% women, with a median age of 36 years (Rq = 13). During mental stress tasks, the median for physiological coherence was similar to baseline coherence but increased significantly with five minutes of directed breathing exercises (38% vs. 63% p <0.0001). The highest perception of stress was during successive subtractions (Me 7, Rq = 4) and the lowest during directed breathing exercises (Me 2 Rq = 3.0). The correlation was sought between physiological coherence and perception of stress during each of the four moments of the study. Basal (Rho Spearman -0.05, p 0.54); Stroop (Rho -0.17, p 0.03); successive subtractions (Rho 0.50, p 0.77); and directed breathing (Rho -0.28, p 0.09).

**Conclusions:**

A correlation was found between physiological coherence and perception of stress during the Stroop test; however, no association was found.

**Disclosure:**

No significant relationships.

